# Application of UHPLC-ESI-Q-TOF-MS to Identify Multiple Constituents in Processed Products of the Herbal Medicine Ligustri Lucidi Fructus

**DOI:** 10.3390/molecules22050689

**Published:** 2017-04-26

**Authors:** Hui Li, Weifeng Yao, Qinan Liu, Jia Xu, Beihua Bao, Mingqiu Shan, Yudan Cao, Fangfang Cheng, Anwei Ding, Li Zhang

**Affiliations:** Jiangsu Collaborative Innovation Center of Chinese Medicinal Resources Industrialization, Jiangsu Key Laboratory for High Technology Research of TCM Formulae, National and Local Collaborative Engineering Center of Chinese Medicinal Resources Industrialization and Formulae Innovative Medicine, and State Key Laboratory Cultivation Base for TCM Quality and Efficacy, School of Pharmacy, Nanjing University of Chinese Medicine, Nanjing 210023, Jiangsu, China; missyhui2012@163.com (H.L.); liuqinan0728@163.com (Q.L.); njxj0106@126.com (J.X.); scotter01@163.com (B.B.); shanmingqiu@163.com (M.S.); raindc@163.com (Y.C.); cff19870524@163.com (F.C.); awding105@163.com (A.D.)

**Keywords:** processed-Ligustri Lucidi Fructus, UHPLC-ESI-Q-TOF-MS, phenylethanoids, flavonoids, secoiridoids, triterpenoids

## Abstract

Ligustri Lucidi Fructus (LLF), the fruit of *Ligustrum lucidum* Ait. (Oleaceae), has been used as a common herbal medicine in clinical practice in China for nearly 2000 years. In most cases, LLF is prescribed in decoctions in the form of processed products rather than crude drugs. In this study, an ultra-high performance liquid chromatography coupled with electrospray ionization-quadrupole-time of flight-mass spectrometry (UHPLC-ESI-Q-TOF-MS) method was established for rapid separation and identification of multiple constituents in the 80% methanol extract of processed-LLF. A total of 50 compounds (one phenylethanoid, seven phenylethanoid glycosides, seven flavonoids, 25 iridoids, nine triterpenoids and one cyclohexanecarboxylic acid) were either unambiguously identified or tentatively characterized with the aid of authentic standards or published data. Luteolin-7-*O*-rutinoside, oleoside and secologanoside were detected in LLF for the first time. This study enriches the chemical profiling of processed-LLF and could provide valuable information for the quality control and further investigation of processed-LLF and crude LLF.

## 1. Introduction

Ligustri Lucidi Fructus (LLF), the dried ripe fruit of *Ligustrum lucidum* Ait. (Oleaceae), also known as Nüzhenzi in Traditional Chinese Medicine (TCM), is not only a widely used herbal medicine in China, but also a functional food material authorized by the China Food and Drug Administration (CFDA). Nüzhenzi was first recorded as a traditional Chinese medicine in the earliest book of Chinese herbal medicine, titled *Shen Nong’s Herbal Classic* [1], which was written in the Western Han Dynasty and has a history of almost 2000 years of use. In this ancient and classical book, Nüzhenzi was listed in the “top class” medicines and claimed to have the ability of maintaining healthy energy. According to the 2015 edition of Chinese Pharmacopoeia, the main function of Nüzhenzi is to nourish liver and kidney, brighten eyes, and make the hair black [2]. Modern research on LLF has demonstrated that various extracts and individual compounds derived from this herbal medicine exhibit extensive bioactivities, such as hepatoprotective, anti-tumor, anti-osteoporosis, antioxidant, anti-inflammatory, anti-ageing, immune regulation, etc. [3]. In clinical practice, crude drugs usually need to be treated in some special way before they can be used as medicines, which is one of the characteristic features of TCMs. In the case of LLF, there are many processing methods, e.g., steaming with wine, vinegar, salt solution or the juice of other herbs, among which, wine steaming is the most important one and has been recorded in the Chinese Pharmacopoeia. After being steamed with wine, the function of LLF will be strengthened and the chemical constituents will change, not only in quantity but also in quality [4]. Over the past few years, several types of chemical constituents have been isolated from LLF, including phenylethanoid glycosides (PhGs), flavonoids, iridoids, triterpenoids and other components such as polysaccharides, amino acids, fatty acids and some minor elements [3]. *Ligustrum lucidum* Ait. is widely distributed in China, and is often used for environmental greening in many places, which makes the source of LLF very abundant. In the view of the medicinal, edible and chemical values, LLF has a great development potential. 

Recently, UHPLC-ESI-Q-TOF-MS has become a powerful tool in the characterization of complex natural products and been widely used in TCM research [5,6,7]. ESI is a soft ionization method capable of providing both protonated and deprotonated molecules. Q-TOF-MS is able to combine high sensitivity and mass accuracy for both precursor and product ions, and therefore makes it possible to confirm the elemental composition for both parent and fragment ions in a rapid and efficient way [8]. Meanwhile, the application of UHPLC can provide high resolution for the separation of complicated natural products and improve the sensitivity of a Q-TOF-MS detector [9]. 

To the best of our knowledge, some constituents, e.g., iridiod glycosides, have been identified in LLF and analyzed using ESI-Q-TOF-MS [10]. However, the comprehensive identification of multiple constituents in processed-LLF has not been reported. Therefore, we decided to investigate the multiple constituents in processed-LLF through UHPLC-ESI-Q-TOF-MS, thus providing in-depth knowledge of its chemical composition and offering valuable information for its quality control and further study.

## 2. Results and Discussion

### 2.1. UHPLC-ESI-Q-TOF-MS Analysis of Constituents in Processed-LLF

**Figure 1:**
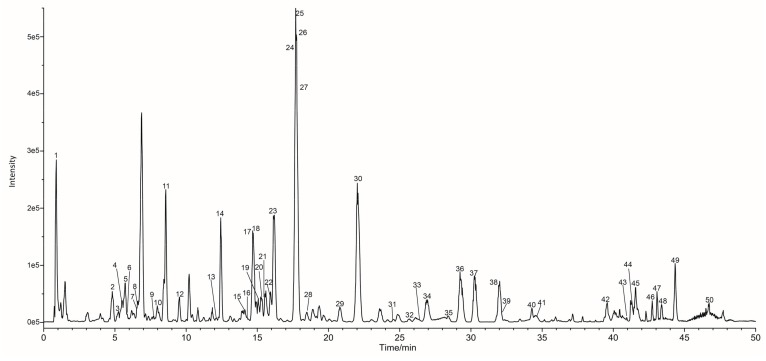
Base Peak Chromatogram (BPC) in negative mode of processed-LLF.

**Figure 2:**
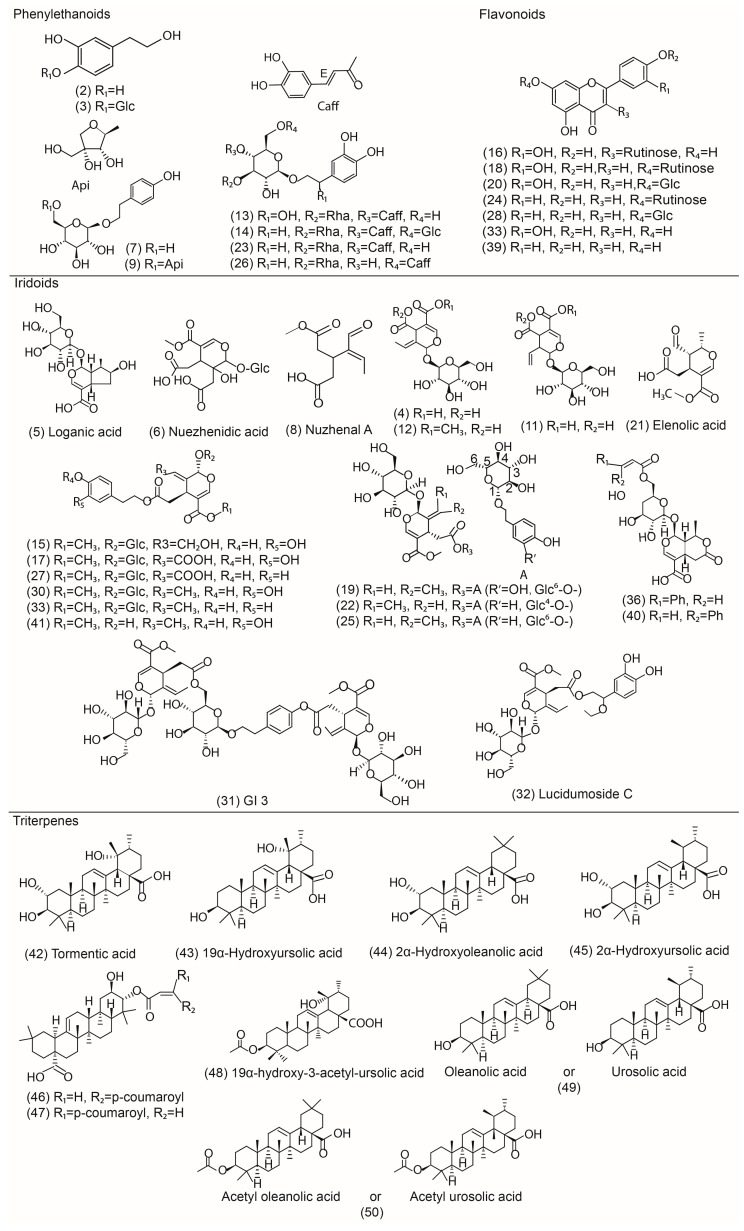
Chemical structures of main compounds identified from processed-LLF. (Glc: β-d-glucopyranosyl; Rha: Rhamnosyl; Caff: Caffeyl; Api: β-d-apiofuranosy).

In this study, most of the compounds showed higher response in the negative mode than in the positive mode. Therefore, negative ion mode has been employed to identify the corresponding signals. The identification of compounds was carried out by comparing their retention times and mass spectra provided by TOF-MS with those of authentic standards when available. The remaining compounds, for which no commercial standards available, were characterized by the interpretation of their mass spectra and the information previously reported in the literature. Figure 1 illustrates the base peak chromatogram (BPC) of the processed-LLF extract in ESI negative mode. As shown in Table 1 and Figure 2, a total of 50 compounds were either unambiguously identified (six compounds) or tentatively characterized (44 compounds).

### 2.2. Identification of Phenylethanoids and Glycosides ***2**, **3**, **7**, **9**, **13**, **14**, **23**, **26***

**Figure 3:**
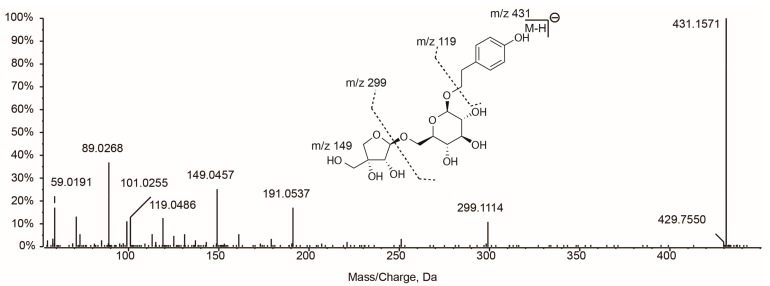
TOF-MS^2^ spectrum and fragmentation scheme proposed for osmanthuside H.

Phenylethanoid glycosides (PhGs) are an important class of compounds in LLF. In this study, one phenylethanoid together with seven PhGs were detected in the 80% methanol extract of processed-LLF. Hydroxytyrosol (**2**) was detected at *m*/*z* 153.0556, with a fragment at *m*/*z* 123, which is due to the loss of the CH_2_OH group [11]. The presence of this phenylethanoid in LLF has been previously reported [35]. Hydroxytyrosol glucoside (**3**) was then eluted with a precursor [M − H]^−^ ion at *m*/*z* 315.1089 and fragmented in MS^2^ analysis to generate main ions at *m*/*z* 123 and 153 [11]. Compound **7** was easily identified as salidroside by comparing its retention time and MS^2^ spectrum with a commercial standard. Salidroside is a glucoside of tyrosol and it′s responsible for the anti-osteoporosis and anti-oxidation actions of LLF [35,36]. According to our previous study, the content of salidroside increased a lot after the LLF was processed. Verbascoside (**23**), isoverbascoside (**26**), β-hydroxyverbascoside (**13**) and echinacoside (**14**) form a class of PhGs with caffeyl groups, thus the MS^2^ analyses of these four compounds showed the same fragments (*m*/*z* 161, 135 and 179) characteristic of the caffeyl group [5,13]. Compounds **23** and **26** were only positional substitution isomers, and their fragmentation patterns were almost the same. Fortunately, compound **23** could be accurately identified as verbascoside by comparing its retention time with a standard. Therefore, compound **26** could be then proposed as isoverbascoside. Osmanthuside H (**9**) was detected at *m*/*z* 431.1558 with a major fragment at *m*/*z* 299, corresponding to the neutral loss of an apiofuranosyl group (132Da) from the precursor [M − H]^−^ ion. The ion at *m*/*z* 299 continued to produce a fragment at *m*/*z* 119 by the loss of the glucoside. Osmanthuside H had been previously reported in LLF [12] and the proposed fragmentation scheme is shown in Figure 3.

### 2.3. Identification of Flavonoids ***16**, **18**, **20**, **24**, **28**, **33**, **39***

As many as eight flavonoids, including six flavones, one flavonol and one isoflavone, were identified in the 80% methanol extract of processed-LLF. Luteolin-7-*O*-glucoside (**20**) was detected at *m*/*z* 447.0934, with a base peak at *m*/*z* 285 in its MS^2^ spectrum, corresponding to free luteolin. In addition, free luteolin (**33**) was also extracted with main MS^2^ fragments at *m*/*z* 133 and 151, which were the result of a notable Ret-Diels-Alder (RDA) cleavage. The presence of this flavone and its glucoside was confirmed using commercial standards. Compound **39** was definitely identified as apigenin by comparison with the authentic substance. Compound **28** exhibited a precursor [M − H]^−^ ion at *m*/*z* 431.0983 with MS^2^ fragments at *m*/*z* 268 and 269, indicating the existence of apigenin aglycone [17]. This compound was thus tentatively identified as apigenin-7-*O*-glucoside (cosmosiin), which had been reported previously in *Ligustrum lucidum* Ait [37]. Compounds **16**, **18** and **24** presented precursor ions at *m*/*z* 609.1466, 593.1511 and 577.1557, respectively, and strong signals for the aglycones were observed in their MS^2^ spectra (**16**: *m*/*z* 301; **18**: *m*/*z* 285; **24**: *m*/*z* 269) produced through the loss of a rutinosyl moiety (308 Da). By comparing their molecular formulas and fragmentation patterns with those reported in the literature [8,14,16], compounds **16** and **24** could be tentatively identified as quercetin-3-*O*-rutinoside (rutin) and apigenin-7-*O*-rutinoside, respectively, both of which had been isolated from *Ligustrum lucidum* Ait. [37,38]. As for compound **18**, the ion at *m*/*z* 285 could be assigned as luteolin or kaempferol aglycone. According to reference [15], luteolin-7-*O*-rutinoside eluted before luteolin-7-*O*-glucoside, while kaempferol-3-*O*-rutinoside eluted after luteolin-7-*O*-glucoside under reversed phase liquid chromatography (RP-LC) conditions. Luteolin-7-*O*-glucoside had been accurately proposed for compound **20** (RT = 15.28 min). Considering the shorter retention time (RT = 14.78 min), compound **18** could be thus tentatively identified as luteolin-7-*O*-rutinoside, which had been already reported in other plants belonging to the genus *Ligustrum*. whilst it is reported here for the first time in LLF [39].

### 2.4. Identification of Iridoids ***4**−**6**, **8**, **10**−**12**, **15**, **17**, **19**, **21**, **22**, **25**, **27**, **29**−**32**, **34**−**38**, **40**, **41***

In most cases, the term iridoid is used to name a wide group of monoterpenes as well as glucoside derivatives, whose structure is in the general cyclopentanopyran form. Cleavage of a bond in the cyclopentane ring of iridoids gives rise to a subclass known as secoiridoids. LLF represents a rich source of iridoids, including cyclopentane iridoids and secoiridoids [3]. As many as 25 compounds of this type were present in processed-LLF, including 24 secoiridoids and one cyclopentane iridoid (loganic acid).

Loganic acid (**5**) was detected by a precursor [M − H]^−^ ion at *m*/*z* 375.1300, and its MS^2^ spectrum gave ions at *m*/*z* 331, 169 and 151. The fragment at *m*/*z* 331 could be justified by decarboxylation from the precursor ion, while the other two fragments represented the continued loss of glucose moieties and subsequent dehydration, respectively [11,19]. Compounds **4** (RT = 5.50 min) and **11** (RT = 8.57 min) were both detected at *m*/*z* 389.1093 with almost the same fragmentation profile (*m*/*z* 345, 227, 209, 183, 165 and 121). The fragment at *m*/*z* 227 was due to the loss of a hexose residue (162Da), which subsequently formed the fragment at *m*/*z* 183 by a neutral loss of CO_2_. The fragment at *m*/*z* 183 could gave ions at *m*/*z* 165 and 121 by successive loss of H_2_O and CO_2_. The above information indicated the presence of oleoside isomers [13,18], which had two carboxylic groups and a hexose in their structure. Oleoside and secologanoside had been reported in the genus *Ligustrum* [40]. Based on high mass accuracy (<5 ppm) and the typical fragmentation profile, Compound **4** was tentatively identified as oleoside, while compound **11** was proposed as secologanoside, which was shown to elute after oleoside under RP-LC conditions [41]. Two isomers **10** and **12** exhibited a deprotonated ion at *m*/*z* 403.1246, corresponding to C_17_H_24_O_11_. Identical fragmentation profile (*m*/*z* 223, 179, 119 and 89) were obtained by ESI-Q-TOF-MS^2^, indicating the presence of oleoside 11-methyl ester and its isomer [8,11,22], which had been previously reported in LLF [42]. Compound **21** was tentatively identified as elenolic acid, based on the intense ion at *m*/*z* 241.0724 and a strong signal at *m*/*z* 139 in its MS^2^ spectrum [27,28].

Compound **30** exhibited a pseudomolecular ion at *m*/*z* 539.1773 with several fragments at *m*/*z* 377, 307, 275, 223, 149, and 139, which were characteristic for oleuropein [8,11,29]. As far as we knew, oleuropein had been isolated from LLF and showed strong antioxidant effects on free radical induced hemolysis of RBC [43]. Besides, oleuropein aglycone (**41**) was also observed by ESI-Q-TOF-MS with a precursor ion at *m*/*z* 377.1243 and products ions at *m*/*z* 307, 275, 149 and 139 [27,29,32]. Other oleuropein derivatives such as oleuropeinic acid (**17**) and hydroxyloleuropein (**15**) were also found in the 80% methanol extract of processed-LLF [8,23,24,25]. In their MS^2^ spectra, they both gave a base peak at *m*/*z* 151, which could be assigned to the phenolic moiety. Compound **34** showed a pseudomolecular ion at *m*/*z* 523.1820 with fragments at *m*/*z* 361, 291 and 259. The fragment at *m*/*z* 361 was due to the loss of glucose, while the other two fragments were produced by the successive loss of C_4_H_6_O and CH_3_OH. These fragments were all 16 mass units less than those of oleuropein, indicating the structure of compound **34** was short a hydroxyl group compared to oleuropein. Based on the fragments pattern and available references [8,10,31], compound **34** was thus tentatively identified as ligustroside. Furthermore, a ligustroside derivative named ligustrosidic acid (**27**) was also detected in processed-LLF, and both of the two compounds had been already reported in LLF [3,42]. 

**Figure 4:**
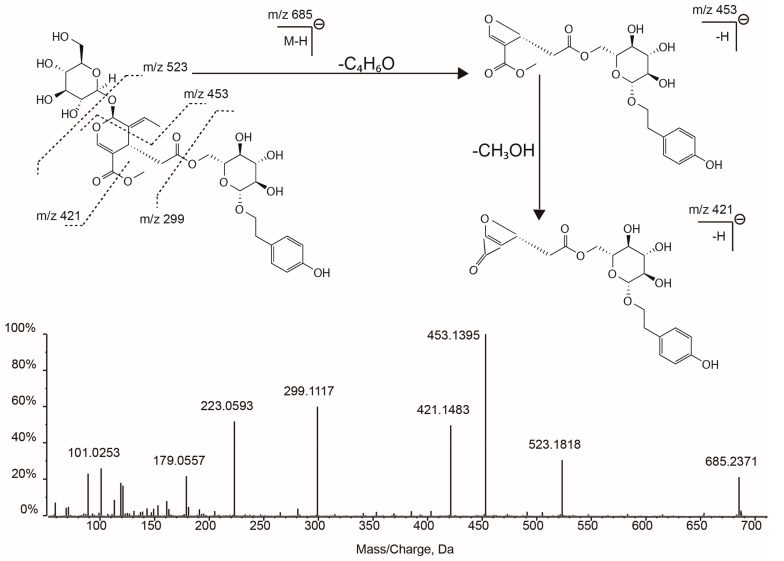
TOF-MS^2^ spectrum and fragmentation scheme proposed for specnuezhenide.

Specnuezhenide (syn. nuezhenide, **25**) is known as one of the most abundant constituents in LLF, and typical fragments (*m*/*z* 523, 453, 299 and 223) was observed characteristic for this secoirdiod glucoside [22,44]. The fragment at *m*/*z* 523 was due to the neutral loss of glucoside, while the fragment at *m*/*z* 299 could be assigned as the tyrosol glucoside moiety in its structure (Figure 4). Furthermore, the identification of this compound was totally confirmed by an authentic standard. Two isomers of nuezhenide (compounds **22** and **29**) were found in the 80% methanol extract of processed-LLF and they exhibited an identical fragmentation profile (*m*/*z* 523, 453, 299 and 223). Compound **29** was then tentatively identified as isonuezhenide based on the later retention time than nuezhenide [26], while it was difficult to ascertain the structure of compound **22** due to the absence of available references. Compound **19** showed a strong signal at *m*/*z* 315 in its MS^2^ spectrum, indicating a hydroxytyrosol glucoside moiety in its structure [26]. Combined with other fragments (*m*/*z* 539, 469, and 437), compound **19** can be tentatively identified as neonuezhenide which owns an extra hydroxyl group than nuezhenide. Compound **32** was tentatively identified as lucidumoside C based on the high accuracy *m*/*z* value at 583.2035 with a ppm as low as 0.5, and the typical fragmentation pattern (*m*/*z* 537, 403, 223 and 151) was in accordance with [8]. Neonuezhenide (**19**) and lucidumoside C (**32**) are two reported antioxidative glucosides from LLF, and they both showed strong antioxidant effects on free radical induced hemolysis of red blood cells [43]. 

The Extracted Ion Chromatogram (EIC) at *m*/*z* 1071.3562 displayed four peaks (**31**, **35**, **37**, **38**) with almost the same fragmentation profiles (*m*/*z* 909, 839, 685, 523, 453, 403, 385) characteristic for G13 and its isomers [13,30], which were possibly nuezhenide with an additional oleoside 11-methyl ester moiety. The fragments at *m*/*z* 685 and 403 could be assigned to the deprotonated ions of nuezhenide and oleoside 11-methyl ester, respectively. G13 was once isolated and identified from LLF, and it was reported to be responsible for the antiosteoporotic bioactivity of LLF [35]. Under RP-LC conditions, G13 was shown to be eluted after oleuropein but before ligstroside [45]. Based on above information, G13 could be proposed as compound **31**. Three candidates, compounds **35**, **37** and **38**, were selected for these G13 isomers, i.e., oleonuezhenide, ligusides A and B, respectively [46]. However, it is impossible to distinguish between them without the use of other analysis methods (e.g., NMR). 

In addition to the above mentioned secoiridiods, another four were also found in processed-LLF, i.e., nuezhenidic acid (**6**), nuezhenal A (**8**), 6′-*O*-*trans*-cinnamoyl-8-epikingisidic acid (**36**) and 6′-*O*-*cis*-cinnamoyl-8-epikingisidic acid (**40**). Nuezhenidic acid (**6**) is a reported secoiridiod glucoside in LLF [20], the content of which was demonstrated to have a significant increase after the LLF being processed with wine [47]. Nuezhenal A (**8**) and the 6′-*O*-cinnamoyl-8-epikingisidic acid isomers **36** and **40** are three recently discovered secoiridiod glucosides from LLF [21], and there are no references about them. These compounds were just tentatively identified base on the high mass accuracy (ppm < 5).

### 2.5. Identification of Triterpenoids ***42**−**50***

Besides the abovementioned compounds, a total of nine triterpenoids were also detected in the 80% methanol extract of processed-LLF. For most of these compounds, only intense precursor ions were obtained by ESI-Q-TOF-MS and it was difficult for the precursor ions to generate more MS^2^ fragments under the conditions used. Thus, the identification of triterpenoids was mainly based on the high mass accuracy (<5 ppm) and the knowledge that these compounds were previously reported to exist in LLF. Triterpenoids are an important type of compounds in LLF, including a pair of isomers named oleanolic acid and ursolic acid which have enjoyed good popularity for their hepatoprotective effects for both chronic liver fibrosis and acute liver injury induced by various chemical substances [48]. The EIC of the standard solution showed one peak for this two compounds with a retention time at 44.31 min. Compound **49** was eluted at 44.35 min and exhibited the same spectrum as the authentic substances, which was thus proposed as oleanolic acid or ursolic acid. Similarly, compound **50** was identified as acetyloleanolic acid or acetylursolic acid due to the 42 unit heavier mass than compound **49**. Compounds **43**, **44** and **45** were tentatively identified as 19α-hydroxyursolic acid (pomolic acid), 2α-hydroxyoleanolic acid (maslinic acid) and 2α-hydroxyursolic acid (colosic acid), respectively. These compounds are three isomers previously reported to exist in LLF [3,33] and their identification was based on their elution order under the RP-LC conditions [49]. Compound **42** was tentatively characterized as tormentic acid [33] also named 2α,19α-Dihydroxyursolic acid, which has an additional hydroxyl group than compounds **43** and **45**. Compound **48** gave a deprotonated ion at *m*/*z* 513.3579 and yielded major fragment ions at *m*/*z* 495 and 453 by elimination of a molecule of H_2_O and CH_3_COOH, respectively. Therefore, compound **48** was tentatively proposed as 19α-hydroxy-3-acetylursolic acid in LLF [34]. In addition, a pair of *cis-trans*-isomers named 3β-*O*-*cis-p*-coumaroyl-maslinic acid and 3β-*O*-*trans-p*-coumaroylmaslinic acid were also detected in processed-LLF, and they were tentatively proposed as compounds **46** and **47**, respectively. The intense signal at *m*/*z* 145 obtained by ESI-Q-TOF-MS^2^ indicated the presence of the coumaroyl moiety, and the two isomers were distinguished by the eluting orders reported in reference [50].

### 2.6. Other Compounds

Finally, a cyclohexanecarboxylic acid identified as quinic acid was also detected in the sample solution.

## 3. Materials and Methods

### 3.1. Chemicals and Reagents

HPLC-grade acetonitrile and formic acid used for LC-MS analysis were supplied by Merck KGaA (Darmstadt, Germany) and Anaqua Chemicals Supply Inc. (Houston, TX, USA), respectively. Deionized water was obtained from a Milli-Q water purification system (Millipore, Bedford, MA, USA). Methanol (HPLC grade) used for sample preparation was purchased from Hanbon Sci. & Tech. (Huaian, China). Standards of oleanolic acid, ursolic acid, luteolin, apigenin, luteolin-7-*O*-glucoside and verbascoside, were purchased from National Institutes for Food and Drug Control (Beijing, China). Salidroside and specnuezhenide were obtained from Chengdu Push Bio-Technology Co., Ltd., (Chengdu, China).

As adjuvant material for crude drug processing, rice wine was purchased from Kuai Ji Shan Shaoxing Wine Co., Ltd. (Shaoxing, China). Rice wine is an alcoholic drink with an alcohol content of 15% (*v/v*), made from rice and traditionally consumed in East Asia, Southeast Asia, and South Asia. There are many types of rice wine, of which Shaoxing wine is probably the best known Chinese rice wine.

### 3.2. Plant Materials and Processing

LLF was purchased from Hetian Chinese Medicine Co., Ltd., (Tongling, China) and identified by Professor Qinan Wu (School of Pharmacy, Nanjing University of Chinese Medicine, Nanjing, China). The crude LLF was processed as follows to generate processed-LLF [51]: 100 g of LLF was put into a stainless steel box, mixed with 30 g of rice wine, steamed over boiling water for 8 h, dried at 50 °C in an air blowing thermostatic oven and then stored in a cool and dry place.

### 3.3. Sample and Standard Solution Preparation

The processed-LLF was crushed into powder and 0.6 g accurately weighed into a 50 mL flask with 25 mL of 80% methanol–water (*v*/*v*). It was extracted in an ultrasonic bath at room temperature for 30 min. The extract was centrifuged at 15,000 rpm for 10 min and the supernatant was used for LC/MS analysis. All eight standards (oleanolic acid, ursolic acid, luteolin, apigenin, luteolin-7-*O*-glucoside, verbascoside, salidroside and specnuezhenide), were dissolved in 80% methanol-water (*v*/*v*) at a concentration of 100 μg/mL to make a standard solution.

### 3.4. UHPLC-ESI-QTOF-MS System and Conditions

Chromatographic analysis was performed on a Shimadzu UHPLC system (Kyoto, Japan) equipped with LC-20AD XR pumps, CTO-20AC column oven and SIL-20A XR auto injector. Components were separated on a Poroshell 120 EC-C18 (2.1 mm × 100 mm, 2.7 μm) at 30 °C with a flow rate of 0.3 mL/min. The mobile phase was composed of 0.1% (*v*/*v*) formic acid in water (solvent A) and acetonitrile (solvent B). The linear gradient elution program was as follows: 0–10 min, 2–15% B; 10–30 min, 15–25% B; 30–35 min, 25–35% B; 35–40 min, 35–75% B; 40–46 min, 75–95% B; 46–48 min, 95–2% B. The injection volume was set at 4 μL.

MS analysis was performed on a triple TOF 5600 System equipped with an electrospray ionization (ESI) interface (AB SCIEX, Concord, ON, Canada). The MS acquisition was operated in both negative and positive ion mode, and mass rang was set at *m*/*z* 50–1500. The parameters were set as follows in negative ion mode: ion spray voltage: −4500 eV; ion source temperature (TEM): 550 °C; declustering potential (DP): −100 eV; curtain gas (N2): 40 psi; atomizing gas (N2): 55 psi; auxiliary gas (N2): 55 psi. Information-dependent acquisition (IDA) was used to trigger acquisition of MS/MS spectra with collision energy (CE) and collision energy spread (CES) fixed at −40 eV and 20 eV respectively. When it comes to the positive ion mode, the conditions were the same but some voltages can change their polarity to positive. Meanwhile, an automated calibration delivery system (CDS) could regulate the MS and MS/MS data automatically.

### 3.5. Data Processing

Data acquisition and processing was carried out using software Analyst, Peak View 1.2 with the application of XIC manager and Formula Finder (AB SCIEX). The SciFinder database (https://scifinder.cas.org) was employed to get the information of previously reported compounds in LLF.

## 4. Conclusions

In this study, a powerful analytical method has been used to characterize 51 compounds (one phenylethanoid, seven phenylethanoid glycosides, eight flavonoids, 25 iridoids, nine triterpenoids and one cyclohexanecarboxylic acid) in the 80% methanol extract of processed-LLF. Four compounds—quercetin-3,4′-diglucoside, luteolin-7-*O*-rutinoside, oleoside and secologanoside—were identified for the first time in processed-LFF. In addition, the MS fragmentation of the compounds were interpreted and summarized in details. Hence, this study offered a reliable reference for the quality assessment and further research of processed-LLF and crude LLF. The established UHPLC-ESI-Q-TOF-MS method can be applied as a template for the systematic analysis of other complicated herbal medicines. Our next work is to search for differences between the compounds identified in crude and processed materials using a chemometric method.

## Figures and Tables

**Table 1:** Identification of 51 constituents of processed-LLF by UHPLC-ESI-Q-TOF-MS/MS in negative mode.

Peak	RT (min)	Formula	[M − H] ^−^			MS^2^ Fragments (Relative Abundance)	Proposed Compound	Ref.
			*m*/*z* theo	*m*/*z* exp	Error (ppm)			
**Phenylethanoids**								
2	4.81	C_8_H_10_O_3_	153.0557	153.0556	−1	123.0456 (100)	Hydroxytyrosol	[11]
3	5.22	C_14_H_20_O_8_	315.1085	315.1089	1.1	153.0554 (94.24), 123.0454 (100)	Hydroxytyrosol Glucoside	[11]
7	6.60	C_14_H_20_O_7_	299.1136	299.1140	1.2	137.0587 (13.60), 119.0500 (83.73), 113.0250 (27.20), 101.0270 (24.53), 59.0194 (100)	Salidroside	standard
9	7.72	C_19_H_28_O_11_	431.1559	431.1558	−0.1	299.1114 (10.82), 149.0457 (25.15), 119.0486 (12.54), 101.0255 (12.87)	Osmanthuside H	[12]
13	12.13	C_29_H_36_O_16_	639.1931	639.1933	0.4	621.1878 (47.63), 459.1506 (10.31), 179.0341 (69.58), 161.0238 (100), 135.0447 (12.63)	β-Hydroxyverbascoside	[13]
14	12.44	C_35_H_46_O_20_	785.2510	785.2517	0.9	623.2239 (39.09), 461.1683 (2.63), 179.0371 (2.79), 161.0236 (35.63), 135.0450 (3.32)	Echinacoside	[5]
23	16.18	C_29_H_36_O_15_	623.1981	623.1989	1.3	461.1669 (35.12), 161.0234 (100), 179.0342 (7.52), 135.0447 (13.66)	Verbascoside	standard
26	17.81	C_29_H_36_O_15_	623.1981	623.1983	0.3	461.1679 (34.39), 161.0235 (100), 179.0340 (6.70), 135.0448 (12.00)	Isoverbascoside	[5]
**Flavonoids**								
16	14.39	C_27_H_30_O_16_	609.1461	609.1466	0.8	301.0356 (39.90), 300.0278 (89.95), 178.9991 (4.59), 151.0033 (4.34)	Quercetin-3-*O*-rutinoside	[8,14]
18	14.78	C_27_H_30_O_15_	593.1512	593.1511	−0.2	285.0407 (100), 284.0327 (9.31)	Luteolin-7-*O*-rutinoside	[15]
20	15.28	C_21_H_20_O_11_	447.0933	447.0934	0.2	285.0397 (100), 284.0320 (44.59)	Luteolin-7-*O*-glucoside	standard
24	17.60	C_27_H_30_O_14_	577.1563	577.1557	−1	311.0559 (0.57), 269.0450 (100)	Apigenin-7-*O*-rutinoside	[16]
28	18.48	C_21_H_20_O_10_	431.0984	431.0983	−0.1	269.0454 (26.47), 268.0369 (100)	Apigenin-7-*O*-glucoside	[17]
33	26.33	C_15_H_10_O_6_	285.0405	285.0408	1.2	151.0033 (14.30), 133.0297 (68.21)	Luteolin	standard
39	32.14	C_15_H_10_O_5_	269.0456	269.0455	−0.2	151.0041 (14.36), 117.0353 (58.84)	Apigenin	standard
**Iridoids**								
4	5.50	C_16_H_22_O_11_	389.1089	389.1093	0.9	345.1190 (3.03), 227.0557 (37.80), 209.0454 (14.54), 183.0660 (87.28), 165.0551 (67.05), 121.0665 (100)	Oleoside	[13,18]
5	5.71	C_16_H_24_O_10_	375.1297	375.1300	0.9	331.1405 (70.23), 169.0872 (3.45), 151.0761 (25.68), 213.0783 (1.16), 113.0250 (39.00)	Loganic acid	[11,19]
6	5.84	C_17_H_24_O_14_	451.1093	451.1092	−0.3	433.0995 (11.52), 271.0457 (22.27), 239.0177 (14.00), 227.0544 (28.26), 195.0296 (70.07), 183.0653 (25.05), 151.0398 (100), 123.0459 (35.06)	Nuezhenidic acid	[20]
8	6.61	C_10_H_14_O_5_	213.0768	213.0777	4.1	183.0666 (100), 151.0768 (54.45), 121.0670 (31.32), 107.0889 (9.07)	Nuzhenal A	[21]
10	8.06	C_17_H_24_O_11_	403.1246	403.1247	0.3	223.0598 (54.55), 179.0555 (26.50), 121.0307 (41.08), 119.0355 (57.87), 113.0242 (34.44), 101.0249 (55.77), 89.0264 (97.55), 59.0190 (100)	Oleoside 11-methyl ester (isomer)	[8,11,22]
11	8.57	C_16_H_22_O_11_	389.1089	389.1093	0.9	345.1192 (29.00), 209.0449 (17.65), 183.0658 (26.15), 165.0552 (41.48), 121.0656 (62.61)	Secologanoside	[13,18,19]
12	9.53	C_17_H_24_O_11_	403.1246	403.1250	1	223.0608 (47.43), 179.0564 (26.79), 121.0290 (25.08), 119.0360 (52.49), 113.0251 (30.06), 101.0266 (56.07), 89.0270 (100), 59.0193 (83.41)	Oleoside 11-methyl ester	[8,11,22]
15	14.12	C_25_H_32_O_14_	555.1719	555.1725	1	323.0771(3.64), 223.0603(7.70), 151.0397(100), 123.0453(11.68)	10-Hydroxyoleuropein	[8,23]
17	14.71	C_25_H_30_O_15_	569.1512	569.1517	0.9	389.0899 (30.33), 209.0452 (46.58), 151.0402 (100), 123.0455 (26.25)	Oleuropeinic acid	[24,25]
19	15.07	C_31_H_42_O_18_	701.2298	701.2311	1.8	539.1797 (12.19), 469.1375 (20.41), 437.1443 (10.17), 315.1081 (100)	Neonuzhenide	[26]
21	15.48	C_11_H_14_O_6_	241.0718	241.0724	2.5	139.0035 (100), 127.0403 (54.59), 121.0295 (27.57), 101.0262 (44.93), 95.0522 (61.86)	Elenolic acid	[27,28]
22	15.94	C_31_H_42_O_17_	685.2349	685.2360	1.6	523.1853 (75.34), 453.1422 (98.87), 421.1519 (53.10), 299.1134 (100), 223.0606 (82.17), 179.0558 (29.48), 119.0371 (31.24)	Nuezhenide (isomer)	--
25	17.72	C_31_H_42_O_17_	685.2349	685.2356	0.9	523.1818 (30.54), 453.1395 (100),421.1483 (49.59), 299.1117 (59.94), 223.0593 (51.80), 179.0557 (21.62), 119.0374 (17.98)	Specnuezhenide	standard
27	17.83	C_25_H_30_O_14_	553.1563	553.1558	−0.8	509.1670 (11.95), 477.1435 (4.16), 391.1015 (8.35), 373.0941 (41.57), 347.1143 (100), 209.0447 (82.67)	Ligustrosidic acid	[10,25]
29	20.80	C_31_H_42_O_17_	685.2349	685.2356	0.9	523.1856 (24.96), 453.1429 (44.64), 421.1518 (22.12), 385.1150 (22.09), 299.1134 (100), 223.0609 (24.93), 179.0555 (20.58), 119.0364 (25.63)	Isonuezhenide	[26]
30	22.06	C_25_H_32_O_13_	539.1770	539.1773	0.5	403.1253 (14.23), 377.1241 (57.51), 307.0813 (91.75), 275.0877 (79.24), 223.0601 (46.21), 179.0563 (19.28), 149.0241 (100), 139.0381 (54.09)	Oleuropein	[8,11,29]
31	24.56	C_48_H_64_O_27_	1071.3562	1071.3581	1.7	909.3186 (10.15), 839.2691 (9.04), 771.2401 (31.56), 685.2395 (36.40), 523.1839 (26.89), 453.1413 (29.53), 403.1255 (18.24), 385.1166 (12.15), 299.1139 (11.67), 223.0605 (23.17)	G 13	[13,30]
32	25.68	C_27_H_36_O_14_	583.2032	583.2035	0.5	537.1649 (31.84), 403.1255 (35.57), 223.0603 (32.74), 151.0401 (100)	Lucidumoside C	[8]
34	26.91	C_25_H_32_O_12_	523.1821	523.1820	−0.2	361.1296 (27.22), 291.0870 (100), 259.0968 (22.10), 101.0260 (20.27)	Ligustroside	[8,10,31]
35	28.41	C_48_H_64_O_27_	1071.3562	1071.3585	2.1	909.3161 (22.61), 839.2736 (16.57), 771.2440 (20.00), 685.2429 (45.75), 523.1855 (35.60), 453.1438 (23.63), 403.1267 (14.76), 385.1145 (6.14), 299.1135 (13.15), 223.0603 (117.03)	G 13 (isomer)	[13,30]
36	29.28	C_25_H_28_O_12_	519.1508	519.1512	0.7	227.0560 (13.80), 189.0557 (34.00), 183.0664 (34.60), 165.0557 (28.12), 161.0610 (100), 147.0457 (63.08), 121.0669 (46.70)	6′-*O*-*trans*-Cinnamoyl-8-epikingisidic acid	[21]
37	30.27	C_48_H_64_O_27_	1071.3562	1071.3578	1.4	909.3159 (15.90), 839.2724 (12.16), 771.2436 (52.95), 685.2404 (100), 523.1850 (63.76), 453.1421 (44.96), 403.1256 (28.24), 385.1157 (15.01), 299.1137 (28.75), 223.0611 (37.36)	G 13 (isomer)	[13,30]
38	31.99	C_48_H_64_O_27_	1071.3562	1071.3583	1.9	909.3147 (38.95), 839.2689 (30.69), 771.2422 (23.33), 685.2387 (90.89), 523.1830 (61.19), 453.1407 (42.67), 403.1256 (25.28), 385.1148 (7.88), 399.1131 (22.25), 223.0604 (30.57)	G 13 (isomer)	[13,30]
40	34.28	C_25_H_28_O_12_	519.1508	519.1510	0.3	475.1626 (29.18), 209.0447 (14.46), 189.0552 (41.69), 183.0654 (19.17), 165.0565 (29.89), 161.0604 (100), 147.0448 (76.02), 121.0667 (42.59)	6′-*O*-cis-Cinnamoyl 8-epikingisidic acid	[21]
41	34.54	C_19_H_22_O_8_	377.1242	377.1243	0.2	307.0800 (39.23), 275.0899 (25.15), 149.0273 (100), 139.0394 (77.64)	Oleuropein aglycone	[27,29,32]
**Triterpenes**								
42	39.55	C_30_H_48_O_5_	487.3429	487.3424	−1	469.3340 (18.52), 423.3282 (15.84)	Tormentic acid	[33]
43	40.97	C_30_H_48_O_4_	471.3480	471.3473	−1.9	453.3402 (60.01), 407.3328 (18.05), 451.3225 (13.04)	19α-Hydroxyursolic acid	[33]
44	41.30	C_30_H_48_O_4_	471.3480	471.3476	−0.8	-	2α-Hydroxyoleanolic acid	[33]
45	41.56	C_30_H_48_O_4_	471.3480	471.3475	−0.9	-	2α-Hydroxyursolic acid	[3]
46	42.73	C_39_H_54_O_6_	617.3848	617.3846	−0.2	145.0292 (14.69)	3β-*O*-*trans-p*-Coumaroylmaslinic acid	[34]
47	43.08	C_39_H_54_O_6_	617.3848	617.3847	−0.1	145.0288 (17.35)	3β-*O*-*cis-p*-Coumaroylmaslinic acid	[34]
48	43.39	C_32_H_50_O_5_	513.3586	513.3579	−1.2	495.3497 (27.14), 453.3390 (3.71)	19α-Hydroxy-3-acetylursolic acid	[34]
49	44.35	C_30_H_48_O_3_	455.3531	455.3526	−1	-	Oleanolic acid/Ursolic acid	standard
50	46.71	C_32_H_50_O_4_	497.3636	497.3632	−0.9	-	Acetyloleanolic acid/Acetylursolic acid	[34]
**Other Compounds**								
1	0.88	C_7_H_12_O_6_	191.0561	191.0569	4.2	173.0447 (11.54), 127.0394 (20.92), 109.0301 (13.44), 93.0359 (67.79), 85.0314 (100)	Quinic acid	[8,19]
